# Ant colony optimization for parallel test assembly

**DOI:** 10.3758/s13428-023-02319-7

**Published:** 2024-01-26

**Authors:** Luc Zimny, Ulrich Schroeders, Oliver Wilhelm

**Affiliations:** 1 Institute of Psychology and Education, Ulm University, Albert-Einstein-Allee 47, 89081 Ulm, Germany; 2 Institute of Psychology, University of Kassel, Kassel, Germany

**Keywords:** Ant colony optimization, Automatic test assembly, Parallel tests, Declarative knowledge

## Abstract

Ant colony optimization (ACO) algorithms have previously been used to compile single short scales of psychological constructs. In the present article, we showcase the versatility of the ACO to construct multiple parallel short scales that adhere to several competing and interacting criteria simultaneously. Based on an initial pool of 120 knowledge items, we assembled three 12-item tests that (a) adequately cover the construct at the domain level, (b) follow a unidimensional measurement model, (c) allow reliable and (d) precise measurement of factual knowledge, and (e) are gender-fair. Moreover, we aligned the test characteristic and test information functions of the three tests to establish the equivalence of the tests. We cross-validated the assembled short scales and investigated their association with the full scale and covariates that were not included in the optimization procedure. Finally, we discuss potential extensions to metaheuristic test assembly and the equivalence of parallel knowledge tests in general.

Short scales of psychological constructs are essential in survey-based research because psychometrically sound short scales make it possible to save time and reduce individuals' workloads without jeopardizing the validity of the measurement at the population level. Multiple parallel short tests that can be used interchangeably are needed if item exposure is a concern or repeated testing is envisioned. Usually, parallel short scales are compiled from a larger pilot tested item pool, making it a task of item sampling targeting predefined goals while considering constraints such as reliability, validity, test fairness, construct coverage, or testing time (Kuhn & Kiefer, 2013; Schroeders et al., 2016a; Spaccapanico Proietti et al., 2020; Steger, Jankowsky, et al., 2022a, Steger, Weiss, et al., 2022b; van der Linden & Glas, 2000; Yan et al., 2014). Different methods have been proposed for this purpose, such as mathematical programming solvers (e.g., Ali & van Rijn, 2016; Becker et al., 2021), machine learning (e.g., Sun et al., 2022), and metaheuristic algorithms (e.g., Leite et al., 2008; Schroeders et al., 2016a).

In the present study, we illustrate the versatility of the metaheuristic ant colony optimization algorithm (ACO; Leite et al., 2008; Marcoulides & Drezner, 2003) for assembling parallel short scales. Prior applications of ACO have focused exclusively on constructing single scales (Jankowsky et al., 2020; Janssen et al., 2015; Olaru & Jankowsky, 2021; Schroeders et al., 2016b, a). We extend previous work by simultaneously compiling three parallel declarative knowledge scales that serve as indicators of crystallized intelligence (*g*_c_; Cattell, 1987), which is an important predictor in many applied settings such as educational achievement (Postlethwaite, 2011; Rohde & Thompson, 2007), job performance (Hunter, 1986), or even death (Deary et al., 2021). We illustrate that, using ACO, we can develop short scales with sufficient construct coverage and that the psychometric attributes of the parallel tests are congruent (i.e., test characteristic curves and test information functions). At the same time, model fit, reliability, and gender fairness can also be optimized. Thus, the present work showcases the flexibility of ACO in parallel test assembly.

## Challenges in test assembly of parallel tests

Once an initial item pool has been developed and validated, researchers are faced with assembling a final test version with a reduced item set that meets several requirements. These requirements are manifold for ability tests, including sufficient reliability (or measurement precision), an appropriate range of item difficulty, decent construct coverage, model fit, fairness, predictive validity, and test duration. Critically, these requirements interact and vary with the selected item set: One item set might be particularly reliable but have weak predictive validity, whereas another is highly predictive but takes too long to complete. Therefore, it is necessary to identify an item set that best combines the desired properties. Testing all possible permutations quickly becomes unfeasible. For example, one can assemble $$\left(\begin{array}{c}30\\ 10\end{array}\right)$$, that is, over 30 million different 10-item tests from an initial item pool of 30 items. The compilation of several parallel test versions is even more complex in terms of the combinatorial complexity of the task.

Going beyond the compilation of a single scale introduces further criteria to consider. From the perspective of classical test theory, parallel tests need to have equal observed-score means, variances, and reliabilities (Lord et al., 2008). In item response theory (IRT), tests are considered weakly parallel when their information functions are identical (Samejima, 1977) and strongly parallel if they have both the same length and identical test characteristic functions (Lord, 1980). Further, in both frameworks, psychological attributes such as content equivalence must also be secured besides psychometric attributes (McDonald, 1999). For example, in the context of knowledge assessment, the same knowledge domains should be covered in equal shares across parallel tests. Importantly, parallel test compilation must jointly consider and align all these criteria from parallel forms. Compiling tests manually from a large item pool while simultaneously considering multiple intertwined psychometric and psychological criteria is unfeasible and will almost invariably lead to suboptimal solutions. Therefore, algorithmic approaches have been applied to solve this combinatorial optimization problem.

## Automated test assembly

Automated test assembly (ATA; van der Linden, 2005) refers to applying algorithmic methods to assemble tests that meet pre-specified criteria. The most prominent methods are mixed integer linear programming (MILP; van der Linden, 2005) and metaheuristic algorithms (e.g., Chang & Shiu, 2012; Leite et al., 2008; Schroeders et al., 2016a; Veldkamp, 1999). All methods aim to solve constrained combinatorial optimization problems efficiently.

MILP is a mathematical optimization technique commonly applied for ATA (see van der Linden, 2005, 2015, for an overview and introduction). It requires the formulation of a list of test specifications that comprise formalized quantitative (e.g., reliability, difficulty), categorical (e.g., item content), or logical attributes (e.g., item overlap). The requirements are formulated as linear functions that are integrated into a common objective function, which is then optimized by a mathematical solver to find an optimal solution (van der Linden, 2015). There is a substantial body of research on MILP, and MILP is routinely applied in large-scale educational assessments (e.g., Becker et al., 2021; Kuhn & Kiefer, 2013; OECD, 2019). In recent years, the solvers have become highly efficient in handling large-scale test assembly problems (Koch et al., 2022). However, MILP also has two significant drawbacks. First, objective functions and constraints are almost exclusively formulated at the item level (van der Linden, 2015). While certain test-level characteristics are influenced by item-level characteristics (such as average test difficulty or test information at a specific ability level), this relationship does not apply to some crucial criteria researchers frequently focus on. For example, measures of overall model fit (e.g., comparative fit index [CFI], root mean square error of approximation [RMSEA]) cannot be estimated from pre-computed item-level indices. Second, MILP is technically and conceptually challenging, making it difficult to apply for researchers that are not trained in linear programming, even though few worked examples and free software packages have been published (Becker et al., 2021; Diao & Van Der Linden, 2011).

Metaheuristic algorithms have gained popularity as versatile tools for ATA (Leite et al., 2008; Schroeders et al., 2016a). Among the plethora of nature-inspired algorithms (Xing & Gao, 2014), the ACO algorithm has often been applied in psychological assessment. ACO is a metaheuristic algorithm inspired by the foraging behavior of ants (Deneubourg et al., 1983). Prior studies have used ACO exclusively for assembling single scales in the confirmatory factor analysis (CFA) framework (e.g., Jankowsky et al., 2020; Kerber et al., 2022; Leite et al., 2008; Olaru & Jankowsky, 2021; Schroeders et al., 2016b; Schultze & Eid, 2018; Steger, Jankowsky et al., 2022a, Steger, Weiss et al., 2022b; Watrin et al., 2019). However, it can also be applied to assemble multiple parallel scales in the IRT framework. With regard to potential drawbacks of ACO, the algorithm may be less efficient than MILP, and the results depend on hyperparameter tuning. The following briefly explains how ACO works and how to assemble short scales that adhere to multiple criteria (see also Olaru et al., 2019, for an introduction).

## Ant colony optimization

**Fig. 1:**
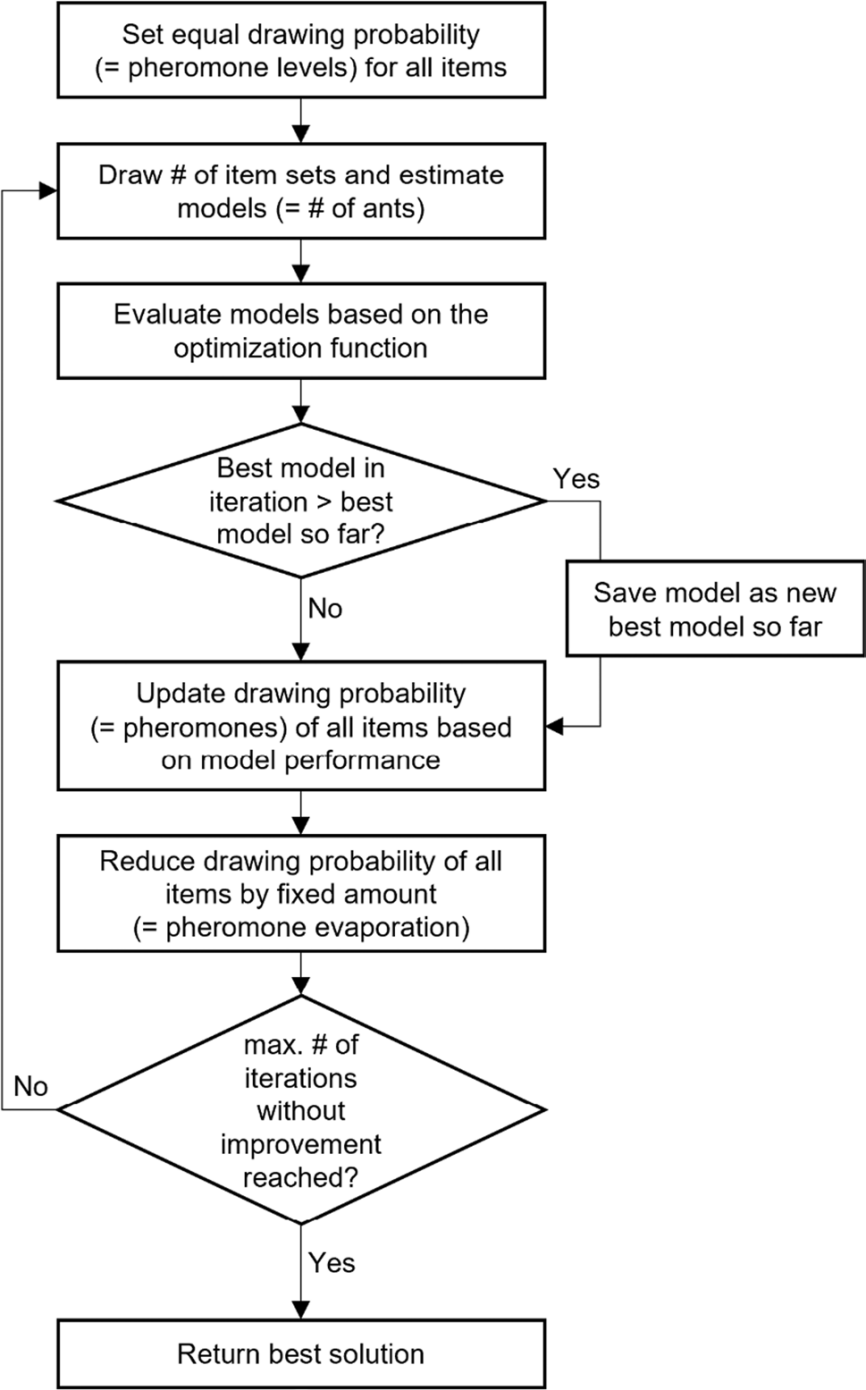
Flow chart of ant colony optimization (ACO) algorithm

The technical details of the ACO algorithm have been comprehensively described elsewhere (e.g., Deneubourg et al., 1983; Dorigo & Stützle, 2019; Marcoulides & Drezner, 2003; Olaru et al., 2019). Therefore, we provide a conceptual introduction to the algorithm within the context of ATA. Figure 1 provides a flow chart of the ACO algorithm: At the outset, all items in the item pool have equal drawing probabilities corresponding to virtual pheromone levels. ACO begins by selecting multiple random item sets, mimicking multiple ants searching different routes to find the shortest path to a food source. Subsequently, all models are evaluated with respect to an optimization function. This function addresses the targeted criteria (e.g., maximizing reliability, maximizing model fit, minimizing differential item functioning). After each iteration, ACO checks whether any model (= ant) is better than the current best model. If so, it saves this model as the new best model. Subsequently, the drawing probability of items included in the best model is increased. Thereby, the probability of drawing an item set that satisfies predefined criteria increases over iterations. This procedure mimics the phenomenon of more pheromones accumulating on shorter (i.e., better) routes. In contrast, a process referred to pheromone evaporation reduces the drawing probability of all items by a small, fixed amount after each iteration (e.g., 5% per iteration). This avoids prioritizing initially drawn item sets or, put differently, avoids local optima in the iterative search. Thus, the algorithm balances between *intensification* (i.e., further refining already good solutions) and *diversification* (i.e., widening the scope to enable a broad exploration of the solution space; Blum & Roli, 2003). ACO iteratively performs the steps of sampling, evaluation, pheromone updating, and evaporation until it finds an item set that fulfills all criteria or reaches a certain number of iterations without further improvements. The procedure remains the same for parallel test assembly, but instead of sampling and evaluating a single item set, multiple item sets are drawn. Their criterion values can then enter the optimization function individually or jointly.

## The present study

In the present study, we illustrate the utility and versatility of ACO for automatic assembly of parallel tests. To that end, we simultaneously draw three 12-item short declarative knowledge scales from a pool of 120 knowledge items. We chose 12 items to retain the construct coverage of 12 knowledge domains as in the full scale. The number of parallel tests is not crucial here and mainly serves for purposes of illustration, as do the requirements defined below. They do not affect the functioning of the algorithm, and their specific instantiation ultimately depends on the specific purpose of the assembled parallel tests. The requirements for the short scales are content-related and psychometric, and they refer to both the individual tests and the equivalence across parallel test versions. As a content-based requirement, we target a broad construct coverage of declarative knowledge across 12 domains (e.g., chemistry, law, arts). Regarding psychometric criteria for the individual tests, we aim to select short scales that adhere to unidimensional measurement models and provide sufficiently reliable total scores for population-level analyses. Further, we aim to sample item sets that show near-zero gender differences at the test level. Regarding the equivalence of the parallel test versions, we minimize the difference in test information and test characteristic curves to ensure that the three short scales are comparable in difficulty and precision. Moreover, we substantiate the equivalence of the short scales by investigating their relations to several covariates (e.g., age, interest).

## Method

### Samples and measures

The study was conducted according to the ethical guidelines for online studies of the German Society for Online Research (DGOF) and in accordance with the Declaration of Helsinki. Ethical approval was not required as per local legislation. Participants were recruited via a German online panel and provided informed consent. Comprehensive sample descriptions have been previously detailed in publications that rely on the same data set (Schroeders et al., 2021; Watrin et al., 2022). For the present study, we only included participants who indicated their gender as either men or women, resulting in a sample size of 1607 participants. The data set was split randomly and in equal shares into a training sample in which the short scales were derived using ACO and an independent testing sample to cross-validate the key parameters of the scales (de Rooij & Weeda, 2020).

To provide further evidence for the psychometric soundness of the assembled parallel scales, we administered two of the three final short scales to an independent large replication sample. The sample comprised both graduate students and the general population and was tested online as part of other investigations. Table 1 provides an overview of the demographic variables for all subsamples of the present study.

**Table 1:** Demographic variables of the training, validation, and replication samples

	Training	Validation	Replication
*N*	803	804	3634
Women	49.1%	45.8%	53.9%
Age,* M* (*SD*)	44.8 (14.6)	45.7 (14.7)	33.1 (12.6)
Education			
None	0.1%	0.1%	0.5%
Elementary school	4.9%	5.2%	17.7%
Intermediate track school	19.4%	18.8%	22.4%
Academic track school	15.4%	15.8%	39.0%
Vocational training	28.1%	27.7%	7.8%
University degree	31.9%	32.1%	12.6%

A comprehensive knowledge test with 120 items (hereafter referred to as “full scale”) constitutes the item pool from which the three short scales were sampled. The knowledge test covers four broad areas of knowledge (social sciences, natural sciences, life sciences, humanities) and 12 domains (e.g., law, chemistry, medicine, arts). Each knowledge domain was measured with ten multiple-choice items (for more information, see Watrin et al., 2022). In addition to the German knowledge test, participants completed a 12-item measure of openness/intellect (Olaru et al., 2015) and a 30-item measure of interests (Armstrong et al., 2008). All items are openly available at https://osf.io/u68nk/.

### Ant colony optimization function

For the parallel scales, we targeted (a) adequate construct coverage, (b) good model fit of a unidimensional model, (c) sufficient reliability at the population level, (d) equal precision, (e) equal difficulty across test versions, and (f) fair measurement across gender groups (see Table 2). In the following, we first describe an empirical approach to determine the thresholds of these criteria. Next, we explain how we included these thresholds in the optimization function of the ACO algorithm.

**Table 2:** Optimization criteria for the three parallel short scales

Criterion	Description
*Construct coverage*	Each short scale comprises one item from each of 12 different knowledge domains (e.g., chemistry, law, art).
*Model fit*	Each short scale conforms to a unidimensional measurement model, as indicated by the comparative fit index (CFI) and the root mean square error of approximation (RMSEA).
*Reliability*	Each short scale is reliable, as indicated by the expected-a-posteriori (EAP) reliability (r_xx_).
*Precision*	The three short scales are equal in precision, as indicated by similarly shaped and located test information functions (TIF).
*Difficulty*	The three short scales are equal in difficulty, as indicated by similarly shaped and located test characteristic curves (TCC).
*Fairness*	None of the short scales exhibits substantial differences between women and men, as indicated by low differential test functioning (DTF).

### Determination of thresholds

Establishing an appropriate target threshold significantly impacts the performance of the item sampling procedure, making it an essential parameter in the application of ACO. Thresholds are often established in advance based on more or less agreed-upon conventions (e.g., the Hu & Bentler, 1999, cutoff values for model fit indices, but see also McNeish & Wolf, 2021, for a criticism of fixed cutoffs). Ideally, the assembled short scales should satisfy all criteria, but the intended cutoffs might be out of reach with a limited initial item pool or with several competing optimization criteria. For example, there is an inherent tension between reliability and validity in psychological scales (Clifton, 2019; Steger, Jankowsky et al., 2022a, Steger, Weiss et al., 2022b). Determining cutoff values a priori can be challenging without prior experience. Also, many criteria are arbitrary to a certain degree, especially if the criterion varies strongly across item or person samples or if the range of the criterion values is unknown (e.g., differences between multiple test information curves).

To overcome these issues, we propose a data-driven method to determine empirical thresholds without prior experience or guidelines (see also Steger, Jankowsky et al., 2022a, Steger, Weiss et al., 2022b). Specifically, the procedure draws random models with a fixed structure (e.g., a unidimensional model with 20 items) to derive an empirical distribution of the targeted criteria. Thresholds can then be set based on percentiles of these distributions (5th or 95th percentiles; see below). By computing percentiles of the parameter distributions, one can determine realistic empirical thresholds (independent of the shape of the distribution) instead of using generic thresholds established in different settings.

**Fig. 2:**
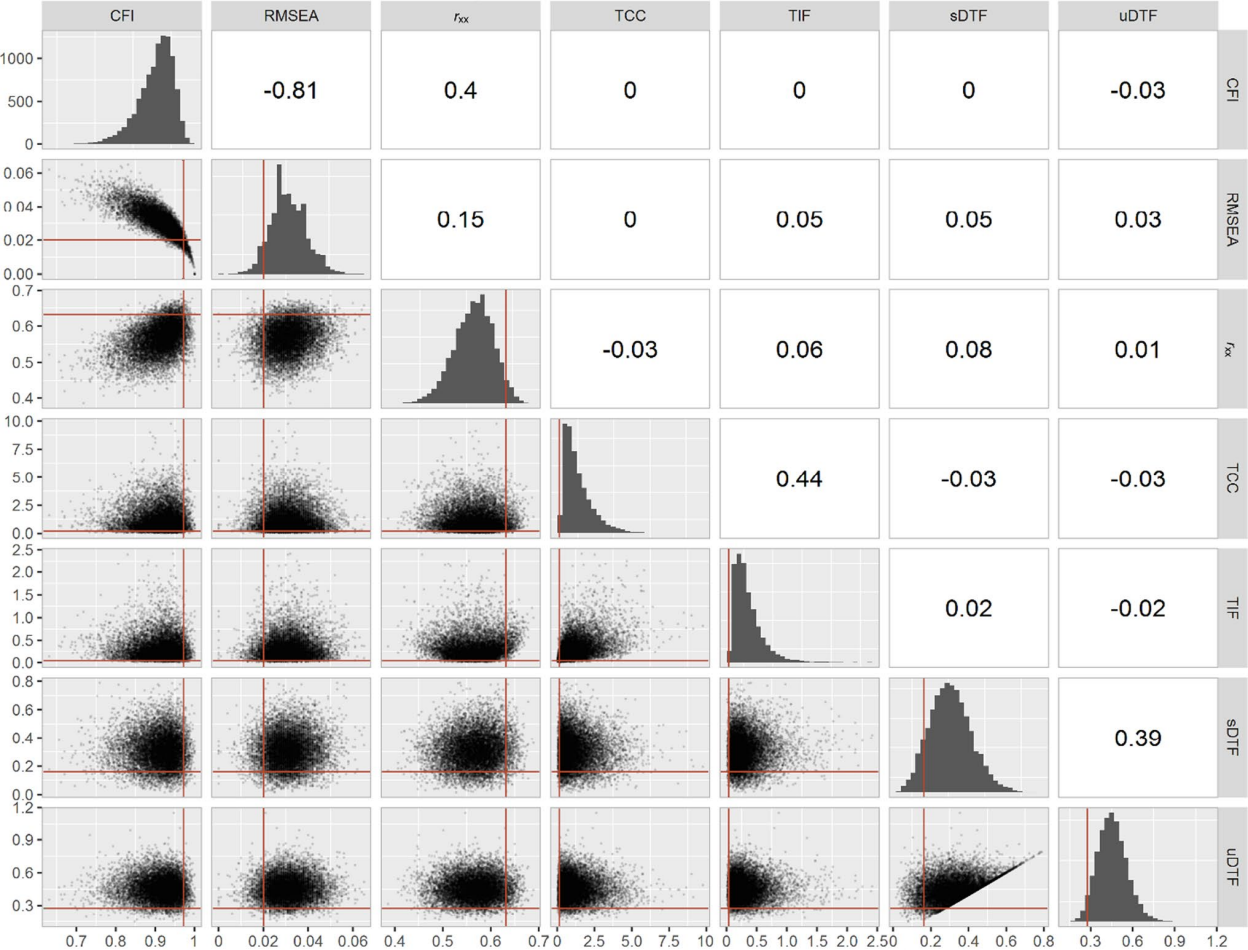
Distributions and correlations of the optimization criteria across 10,000 random samples. *Note*. CFI = comparative fit index, RMSEA = root mean square error of approximation, *r*_xx_ = reliability, TCC = test characteristic curve, TIF = test information curve, sDTF = signed differential test functioning score, uDTF = unsigned differential test functioning score. TCC and TIF values are divided by 1000 for readability. Solid red lines mark the 5th or 95th percentile of the indices' distribution, constituting the empirical thresholds later used in the optimization function

In the present case, we drew 10,000 times three randomly compiled knowledge tests with nonoverlapping item sets. The three models reflect the three parallel test versions. Each test consists of 12 items from the initial pool of 120 knowledge items (Watrin et al., 2022). For each knowledge test, we estimated a unidimensional two-parameter logistic (2PL) model (DeMars, 2010). For each set of three models, we extracted the values of the criteria to be included in the optimization function (see Table 1) to establish their distributions and correlations (see Fig. 2). Finally, we computed the 5th and 95th percentiles based on the distributions. This is evidently a data-driven approach, but we argue that it is more stringent than using uninformative thresholds.

The empirical percentiles provide a threshold that might sensibly be achieved with the available item pool. Figure 2 illustrates the results from the random sampling procedure and the resulting cutoff values of the different criteria based on the 5th (RMSEA, TIF, TCC, DTF) and 95th (CFI, *r*_xx_) percentiles. The random sampling procedure gives valuable insight into redundancies between the optimization criteria. For instance, there was a strong correlation between the measures of model fit (*r*(CFI,RMSEA) = −.81). If both measures were independently included in the optimization procedure, this would effectively result in an overweighting of model fit relative to other optimization criteria. Accordingly, we combined the two fit indices to compute a single criterion for model fit [see formula (3) below]. The same goes for the sDTF and uDTF, which inform about different aspects of DTF and were therefore considered jointly as an emergent variable reflecting overall (un)fairness in the test. The observed correlation between the two indices was moderate (*r*(sDTF,uDTF) = .39), but this correlation is biased downwards due to the censored data distribution (see Fig. 2). As is to be expected, the TIF and TCC were positively correlated (*r*(TIF,TCC) = .44) because the TCC constrains the TIF (van der Linden & Luecht, 1996). However, it is still sensible to consider the indices separately in test assembly because they reflect different aspects of the test, and their correlation is far from unity (Ali & van Rijn, 2016). The other correlations of psychometric criteria did not indicate meaningful redundancy.

In the optimization function, all values were logit-transformed to place them on a common metric between 0 and 1. We used the empirically determined thresholds as the inflection points of the logit functions to maximize differences in the most decisive region (Olaru et al., 2019).

### Determination of slopes

**Fig. 3:**
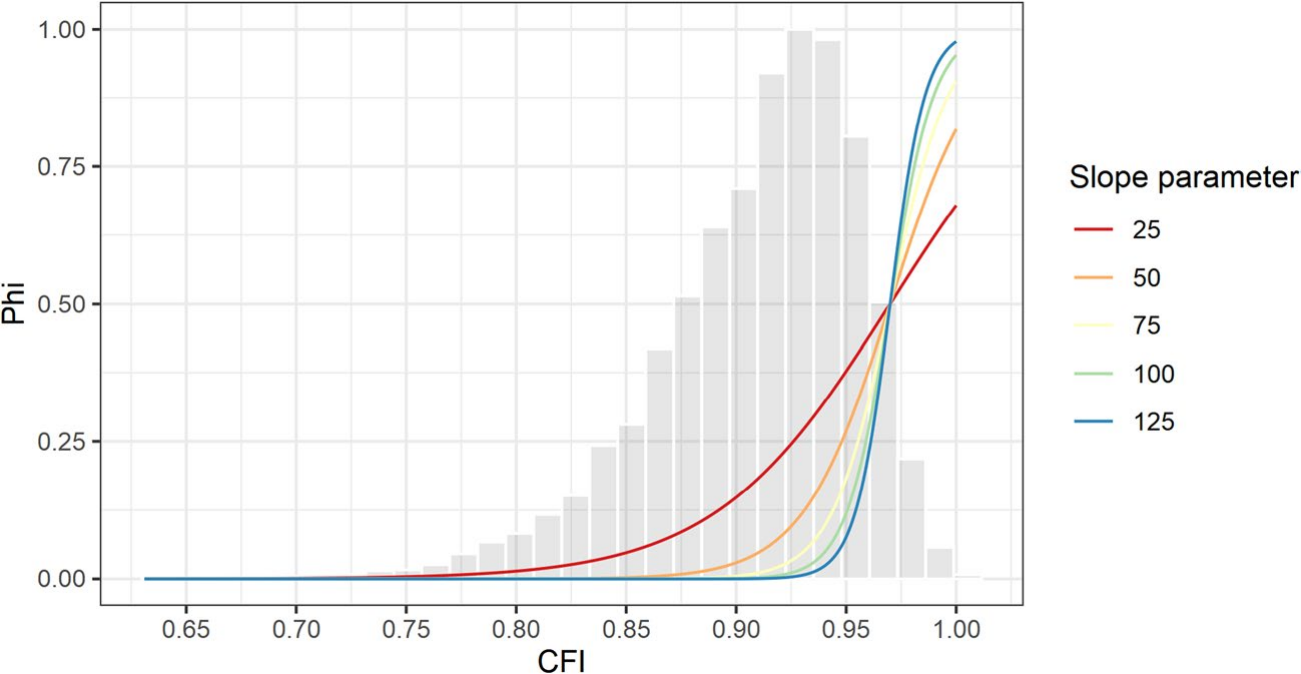
Optimization functions with a fixed threshold and different slopes. *Note*. Histogram of CFI values from 10,000 randomly sampled models. The logit functions share a common threshold of .97, which was established through the random sampling procedure, but they vary in their slope parameter

There is no research yet concerning the optimal choice of slope parameter. Previous studies have mostly optimized standardized criteria ranging between 0 and 1 (e.g., reliability, criterion correlations, model fit). For such criteria, the slope parameter is usually set between 15 and 100 (e.g., Jankowsky et al., 2020; Janssen et al., 2015; Olaru et al., 2019; Schroeders et al., 2016a, b, 2023). In the absence of prior experience, we propose using the results of the random sampling procedure to approximate sensible slope parameters. Figure 3 illustrates the empirical distribution of CFI values from the 10,000 random models combined with five logit functions with the same threshold but different slope parameters. As can be seen, very flat slopes reward a sizable number of models that do not satisfy the required criterion. In contrast, very steep slopes assign low pheromone levels even to models only slightly below the targeted threshold. Given logit functions with varying slope parameters and the results of the random sampling procedure, one can identify the slope that suits the individual needs. For example, in the present study, a slope of 100 resulted in the top 10% of models receiving a pheromone level larger than 0.25. The R code accompanying this article provides functions to visualize the logit function, facilitating a stepwise identification of appropriate slope values. When in doubt, we recommend flatter slopes to ensure that a criterion is considered at all, at the price of longer running times.

### Determination of weights

Like the solvers in MILP, ACO requires a single objective function to optimize. Therefore, once the individual parts of the overall optimization function had been determined [functions (1) to (9)], we created a single overall optimization function [see function (10)]. This is commonly done by summing and optionally weighting individual objective functions (e.g., for model fit or reliability). Extensive research has been conducted on multi-objective optimization (Deb, 2011). Ultimately, assigning different weights expresses priorities in the optimization function (Marler & Arora, 2010). Weights can be increased to emphasize the importance of certain criteria (e.g., model fit) or to address redundancy between criteria (e.g., reducing the individual weights of two highly correlated criteria). In the absence of prior knowledge or experience, we suggest implementing a parsimonious and pragmatic equal-weighting scheme that assigns equal weights to all criteria (e.g., Janssen et al., 2015; Olaru et al., 2019; Watrin et al., 2019).

### Criterion 1: Construct coverage

The full scale with 120 items covered 12 different knowledge domains. To retain adequate construct coverage in the parallel short scales, we imposed the constraint that every short scale must contain exactly one item from each of the 12 different knowledge domains and that these items do not overlap.

### Criterion 2: Model fit

We estimated three unidimensional 2PL models with 12 binary indicators in each iteration and evaluated the CFI and the RMSEA (via the *M*_2_ statistic; Maydeu-Olivares & Joe, 2006). The worst CFI (CFI_min_) and RMSEA (RMSEA_max_) observed in any of the three models was decisive for the respective pheromone level, ensuring that all short scales met the defined requirements. The model fit thresholds established with the random sampling procedure were CFI ≥ .97 and RMSEA ≥ .02, respectively. These values represent the inflection points of the logistic optimization functions [see denominators of functions (1) and (2)].

1 $${\varphi }_{CFI}= \frac{1}{1+ {e}^{100*\left(.97-{CFI}_{{\text{min}}}\right)}}$$

2 $${\varphi }_{RMSEA}= 1-\frac{1}{1+ {e}^{100*\left(.02-{RMSEA}_{{\text{max}}}\right)}}$$

Because the CFI failed to reach the cutoffs more often in the random samples than the RMSEA, we weighted it more heavily in the overall objective function for model fit. This adaptation of the objective function is optional, and the weights are arbitrary. However, it emphasizes the importance of the CFI in the overall optimization and likely increases the probability that ACO finds a suitable solution.

3 $${\varphi }_{Fit}= \frac{3*{\varphi }_{CFI}+1*{\varphi }_{RMSEA}}{4}$$

### Criterion 3: Reliability

We estimated the reliability of the three short scales based on the expected-a-posteriori (EAP) factor scores of the 2PL models (Chalmers, 2012). As for the evaluation of model fit, the lowest reliability (rel_min_) was decisive for the respective pheromone level, ensuring that all short scales met the defined requirements.

4 $${\varphi }_{Rel}= \frac{1}{1+ {e}^{100*\left(.63-{rel}_{{\text{min}}}\right)}}$$

### Criterion 4: Difficulty

To guarantee comparable difficulty across the parallel short scales (Ali & van Rijn, 2016), we computed their respective TCCs and minimized the squared and summed differences between the curves.

5 $${\varphi }_{Diff}= 1-\frac{1}{1+ {e}^{0.025*(202-{TCC}_{{\text{sqsum}}})}}$$

### Criterion 5: Precision

To support comparable precision across the parallel short scales, we computed their respective TIF and minimized the squared and summed differences between the curves.

6 $${\varphi }_{Prec}= 1-\frac{1}{1+ {e}^{0.08*\left(62-{TIF}_{{\text{sqsum}}}\right)}}$$

### Criterion 6: Differential test functioning

To ensure fairness across gender groups, we assessed uniform and nonuniform DTF using the indices proposed by Chalmers et al. (2016). DTF indicates whether there is a scoring bias between the investigated groups and the test level. The signed DTF measure indicates the extent of overall scoring bias across groups, that is, if the reference group scores consistently lower or higher on average than the focal group(s). The unsigned DTF measure reflects the discrepancy between the reference and focal groups(s) test curves, potentially indicating scoring bias at particular ability (theta) levels. The highest DTF of the three parallel versions was decisive for the respective pheromone level.

7 $${\varphi }_{sDTF}= 1-\frac{1}{1+ {e}^{25*\left(0.13-{sDTF}_{{\text{max}}}\right)}}$$

8 $${\varphi }_{uDTF}= 1-\frac{1}{1+ {e}^{25*\left(0.29-{uDTF}_{{\text{max}}}\right)}}$$

9 $${\varphi }_{DTF}= \frac{{\varphi }_{sDTF}+ {\varphi }_{uDTF}}{2}$$

Finally, we aggregated all previous results of the specific functions into a single global value to be optimized via ACO.

10 $${\varphi }_{overall}= \frac{{\varphi }_{Fit}+{\varphi }_{Rel}+{\varphi }_{Diff}+{\varphi }_{Prec}+ {\varphi }_{DTF} }{5}$$

### Statistical analysis

We performed all analyses using R (R Core Team, 2020). We used the packages *mirt* to estimate IRT models (version 1.36.1, Chalmers, 2012) and *lavaan* to estimate CFA models (version 0.6-12, Rosseel, 2012). We used the packages *doParallel* (version 1.0.17, Microsoft Corporation & Weston, 2022) and *foreach* (version 1.5.2, Daniel et al., 2022) for parallel computation. For general data handling, we used packages from the *tidyverse* (version 1.3.2, Wickham et al., 2019), and for descriptive statistics, we used the package *psych* (version 2.2.5, Revelle, 2020). Data and annotated code for all analyses are provided in a repository of the Open Science Framework: https://osf.io/u68nk/. We also provide the items for the full scale and the three short scales. In addition, we provide a web application that allows for easy norm-based evaluation of the short scales: https://psy-diagnostics.shinyapps.io/gc_scaling/.

## Results

Table 3 shows the psychometric properties of the best three short scales in the training, validation, and replication samples. In line with implemented constraints on the construct coverage, each short scale comprised items from 12 different knowledge domains. Across all samples and scales, the model fit indices were close to the targeted (empirical) thresholds of CFI ≥ .97 and RMSEA ≤ .02. Considering test length and coverage, the reliability was adequate and similar across the parallel short scales and the investigated samples. While higher reliability would be desirable for individual diagnostics, there is inevitably a trade-off between construct coverage (i.e., content validity) and reliability (Clifton, 2019; Steger, Jankowsky et al., 2022a, Steger, Weiss et al., 2022b). Reducing the test length to only 12 items came at the price of reduced measurement precision. Therefore, we suggest that researchers requiring a scale with higher reliability use a combined version of two or three short scales. As Table 3 indicates, a combined 36-item scale had good model fit and substantially higher reliability.

**Table 3:** Psychometric properties of the three short scales and a combined 36-item scale

	Scale									
	1			2			3		1 + 2 + 3	
	Train.	Val.	Rep.	Train.	Val.	Rep.	Train.	Val.	Train.	Val.
*M* (*SD*)	.58	.59	.54	.59	.59	.57	.59	.59	.59	.59
	(.21)	(.20)	(.23)	(.21)	(.21)	(.21)	(.21)	(.21)	(.18)	(.18)
CFI	.982	.957	.979	.964	.974	.963	.967	.967	.954	.954
RMSEA	.020	.026	.026	.029	.022	.028	.028	.026	.030	.030
*r*_xx_	.664	.623	.706	.660	.634	.654	.667	.644	.851	.851
sDTF	.046	−.205	−.135	−.020	−.247	−.095	−.066	−.316	−.066	.915
uDTF	.235	.205	.242	.300	.319	.097	.285	.336	−.670	.839

*N*_Training_ = 803, *N*_Validation_ = 804, *N*_Replication Scale 1_ = 2896, *N*_*Replication* Scale 2_ = 1603. Train. = training sample, Val. = validation sample, Rep. = replication sample, M = average proportion correct, SD = standard deviation of proportion correct, CFI = comparative fit index, RMSEA = root mean square error of approximation, *r*_xx_ = reliability, sDTF = signed differential test functioning score, uDTF = unsigned differential test functioning score

**Fig. 4:**
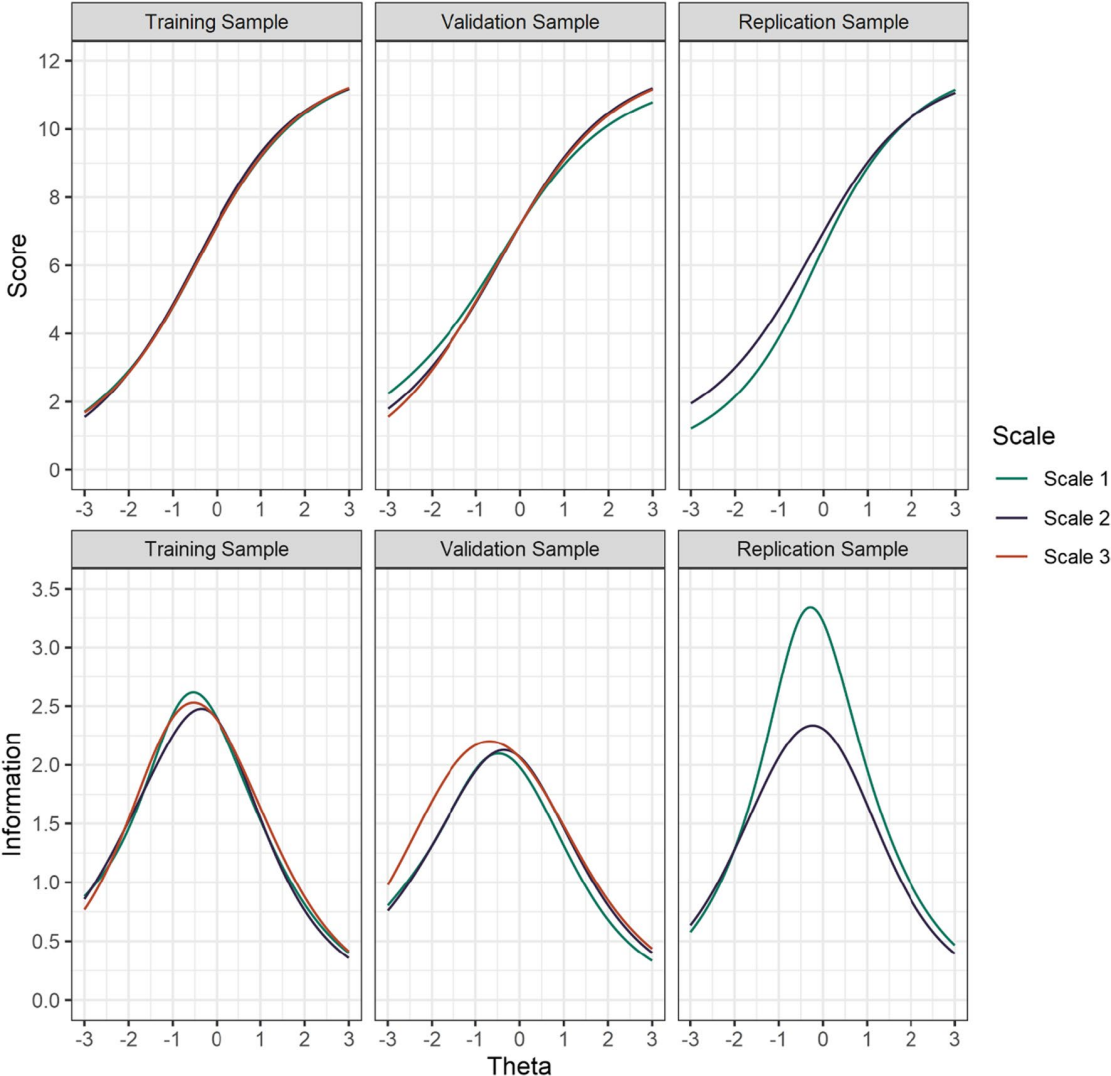
Test characteristic curves and test information curves of the three short scales in the training, validation, and replication samples

The TCCs of the three scales overlapped strongly in the training and validation samples. They showed only minor deviations in the important range between −2 and 2 on the ability distribution *θ*, indicating a strong equivalence in test difficulty across the three short scales (see the upper part in Fig. 4). In the replication sample, the TCCs of scales 1 and 2 overlapped strongly in the ability range above average but increasingly deviated in the ability range below average.

In the training samples, the TIFs were closely aligned, indicating highly comparable information across the entire ability range. In the validation sample, the TIFs were closely aligned for two scales, while one scale (scale 3) discriminated slightly better at lower levels of ability (*θ* < 0.5) than the two other scales. In the replication sample, scale 2 had a similar TIF as in the training and validation samples, and the TIF for scale 1 had a similar distribution across the ability range but was more informative overall.

Concerning gender differences, both the signed and unsigned DTF scores indicated little differential test functioning between women and men. As both measures remain in the original metric (Chalmers et al., 2016), they indicate less than one-third of a point difference in the test scores between men and women on a scale from 0 to 12, or less than one point on the combined scale from 0 to 32, respectively.

### Correlations among scales

In the validation sample, the manifest correlations between the sum scores of the three short scales were substantial (*r*_1,2_ = .62, *r*_1,3_ = .63, *r*_2,3_ = .64). To estimate correlations free of measurement error, we fitted a CFA in which each 12-item set loaded on one latent factor, and the three factors were allowed to correlate freely. This model had satisfactory fit (χ^2^(591, *N* = 804) = 865.1, *p* < .001, CFI = .948, RMSEA = .024), and the latent factors correlated perfectly. This indicates that apart from measurement error, the rank orders of participants are perfectly preserved across the short scales. In fact, the three latent factors correlated slightly above 1 (*r*_1,2_ = 1.03, *r*_1,3_ = 1.04, *r*_2,3_ = 1.01), also known as Heywood case (Savalei & Kolenikov, 2008). This is to be expected with values at the limit of the possible range, and constraining correlations of latent variables to unity did not deteriorate model fit significantly [Δχ^2^(3, *N* = 804) = 3.15, *p* = .37].

### Correlations with covariates

To further substantiate the equivalence and validity of the three short scales, we investigated their association with the full scale and several covariates in the validation sample, namely age, gender (1 = *women*, 2 = *men*), education (1 = *none* to 6 = *university degree*), the personality factor openness (Olaru et al., 2015), and vocational interests (Armstrong et al., 2008). To test for meaningful differences in the correlation coefficients, we computed 95% confidence intervals based on nonparametric bootstraps with 10.000 iterations (Cumming, 2014). Table 4 shows that the 95% confidence intervals did not indicate meaningful differences between the correlation coefficients.

**Table 4:** Correlations of the three short scales and the 120-item full scale with the covariates

	Scale			
	1	2	3	Full scale (120 items)
Full scale	.80 [.77, .82]	.82 [.79, .83]	.82 [.80, .84]	
Age	.12 [.06, .19]	.09 [.03, .13]	.13 [.06, .20]	.12 [.06, .15]
Gender	.06 [−.01, .12]	.06 [−.01, .13]	.10 [.03, .15]	.06 [.01, .11]
Education	.30 [.25, .36]	.34 [.27, .40]	.29 [.22, .34]	.35 [.31, .39]
Openness/intellect	.25 [.21, .32]	.24 [.18, .31]	.22 [.18, .29]	.29 [.24, .33]
Vocational interests				
Realistic	−.11 [−.17, −.05]	−.12 [−.17, −.05]	−.08 [−.14, −.02]	−.09 [−.13, −.05]
Investigative	.12 [.05, .17]	.11 [.05, .18]	.13 [.06, .18]	.18 [.12, .22]
Artistic	.14 [.07, .20]	.11 [.04, .17]	.12 [.06, .17]	.14 [.08, .19]
Social	.09 [.02, .15]	.06 [.01, .14]	.08 [.03, .14]	.11 [.05, .15]
Enterprising	−.10 [−.16, −.04]	−.08 [−.14, −.01]	−.09 [−.16, −.04]	−.09 [−.13, −.03]
Conventional	−.15 [−.21, −.09]	−.21 [−.27, −.16]	−.16 [−.21, −.09]	−.18 [−.22, −.13]

*N*_Training_ = 803, *N*_Validation_ = 804, *N*_total_ = 1607. 95% confidence intervals in parentheses

## Discussion

Short scales of psychological constructs are indispensable in research and applied settings because they save time and reduce individuals' workloads while maintaining the validity of the measurement at the population level. Parallel scales allow for repeated testing and increase test security in unproctored settings. Selecting items manually to derive parallel and psychometrically sound tests almost inevitably leads to suboptimal solutions. It quickly becomes unfeasible depending on the size of the initial item pool, the desired test length, and the number of criteria to fulfill. Compiling parallel short scales of general declarative knowledge is particularly challenging because the construct definition of general knowledge is inherently broad and heterogeneous. Yet it is essential to ensure an adequate level of reliability. Further, it is difficult to establish unidimensional measurement models of declarative knowledge, given the overlap between knowledge domains and within-item multidimensionality (Schroeders et al., 2021). Finally, knowledge tests tend to disadvantage women, which can be remedied by appropriate item compilation (Schroeders et al., 2016b). Meeting all these competing requirements requires consideration during item selection.

We illustrated how the metaheuristic algorithm ACO can help in solving this combinatorial optimization problem. We demonstrated that the algorithm can be used to construct multiple parallel short scales adhering to several competing and interacting criteria. The three assembled general knowledge tests adhered to the criteria of construct coverage, model fit, reliability, equivalent difficulty, information, and (lack of) differential test functioning. As this study is the first application of ACO in parallel test assembly, further experience is needed to better understand which thresholds, slopes, and weighting schemes are appropriate in different application areas and how different criteria interact. In the following, we discuss generalizability, equivalence, and validity issues, the usefulness of the random sampling approach for threshold determination, and possible extensions to parallel test assembly using ACO.

## Generalizability of the results

Generalizability refers to the extent to which research findings apply to variations in items, persons, and methods. In the present context, such variations are based on different items (item sampling), the recruitment of subjects from different populations (person sampling), or the use of different computational procedures (method sampling). We used cross-validation to investigate overfit in our data and used an independent replication sample (see also Dwyer et al., 2018, for the hierarchy of generalizability) to see whether the psychometric properties were identical in a sample that differed in age and education. The present results support the psychometric equivalence across samples. However, the issue of generalizability is not specific to ACO but concerns the validity of psychological assessment in general (Cronbach et al., 1963).

Methodologically, ACO is not bound to any particular framework (e.g., CFA, IRT) or type of criterion (e.g., item-level, test-level). As a general-purpose local search algorithm, it can optimize any objective that can be expressed numerically. In the present study, we estimated 2PL IRT models because they are parsimonious and fit the data reasonably well. However, ACO could also be applied to 3PL models (e.g., Lord et al., 2008), graded response (e.g., Samejima, 1969), Rasch testlet models (e.g., Wang & Wilson, 2005), network models (e.g., Borsboom et al., 2021) or formative models (e.g., Diamantopoulos et al., 2008). All it requires is to extract the desired parameters and set up an optimization function.

For illustrative purposes, we assembled three parallel short scales, as many high-stakes assessments require two versions of the tests to avoid copying from seat neighbors, and longitudinal research requires at least two forms. However, the approach described can theoretically be extended to more parallel versions with different numbers of items. Whether ACO succeeds in finding an optimal solution depends on the size and quality of the initial item pool. Here, ACO is subject to the same pragmatic limitations as MILP or any other test compilation method. Although the initial item pool was limited (120 items), we successfully assembled three parallel short scales. The random sampling procedure we described to derive the thresholds can provide insights into how promising a parallel test composition is given the size and the quality of the initial item pool, the target criteria, and the number of parallel tests and items.

## Equivalence and validity of short knowledge tests

The three knowledge scales exhibited highly similar means, standard deviations, and reliabilities. The latent factor correlations indicated that the rank orders of subjects did not change across the parallel scales once measurement error was accounted for. Further,

the scales correlated similarly with all investigated external criteria. Therefore, the tests can be considered parallel (AERA et al., 2014), and it is sensible to assume that the "forms measure, within acceptable limits, the same psychological function" (Angoff, 1984, p. 86). However, two important points must be considered.

First, the reliability of the short scale is adequate at the population level, but the measurement precision at the individual level is necessarily low, with only a few heterogeneous items (Mellenbergh, 1996). As the reliability is comparable to other brief cognitive measures applied in survey research (e.g., Schmiedek et al., 2022), and measurement error can and should (Bollen, 2002) be addressed with latent variable models, the scales are well suited for population-level analyses (e.g., analyses of covariance). In turn, confidence intervals around point estimates will be large, introducing considerable uncertainty in individual-level decisions (Kruyen et al., 2013). In cases where measurement precision is crucial, we recommend using the combined scale with 36 items to achieve sufficient measurement precision, or other more comprehensive knowledge tests (e.g., Amthauer et al., 2001; Liepmann & Beauducel, 2010; Watrin et al., 2022; Wilhelm et al., 2014).

Second, given the broad definition of *g*_c_ (Cattell, 1987; Horn & Blankson, 2005; Schneider & McGrew, 2018), a 12-item measure can merely be considered a proxy. In fact, even the 120-item knowledge test, which served as a basis for the short scale compilation, theoretically does not adequately reflect the breadth and depth of *g*_c_, and even broader assessments with thousands of knowledge items, as illustrated by Steger et al. (2019) ; Buades-Sitjar et al. (2021), might be necessary. Indeed, the latent variable *g*_c_ extracted from a specific item set should not be confused with the construct *g*_c_ (Borgstede & Eggert, 2023). The equivalence of the three knowledge scales only applies to the latent variables that capture the common variance of their respective 12 items. At the item level, equivalence in knowledge assessment might be an elusive fiction. For example, the three economy items cover different aspects of the knowledge domain (subsidy, social markets, and outsourcing), and different individuals might have had different learning opportunities for them. Recent studies have shown that items comprise knowledge-irrelevant variance, e.g., age-related (Schroeders et al., 2021) or country-related (Watrin et al., 2023) effects at the item level. While these results do not detract from the utility of total scores, they prohibit item-level comparisons of the parallel scales. The scales assembled in this study are well suited as economic proxies and can serve as informative predictors and covariates in various contexts. However, they are only incomplete measures of *g*_c_.

## The utility of empirical thresholds

The proposed random sampling approach helps identify redundancies between criteria and sets achievable thresholds for each criterion. This is important because we know little about how different test characteristics covary in real-world data. While theoretical associations between test characteristics are well established, their interaction is rarely considered comprehensively. Given the plethora of variables that might affect such interactions, it is difficult to derive overarching rules of thumb that hold in vastly different settings. Redundancies (i.e., high correlations) between criteria are problematic because they lead to a relative overweighing and, thus, unbalanced item selection. For example, the CFI and RMSEA in our study were highly correlated in the random samples because both assess (different aspects of) model fit. Including them separately in the optimization function would have resulted in an almost twofold higher weight for model fit. Therefore, we pooled the estimates. Other redundancies between criteria of interest are not always obvious (e.g., item discrimination and factor loadings). Therefore, the approach we propose is to examine the criteria in a large number of random models to study the empirical interdependencies between different criteria. Obviously, these considerations are derived from a random sample of parallel test compilations, and we need to assume that mutatis mutandis relations between criteria stay the same during ACO estimation. Future studies might also consider the normalization of optimization criteria prior to computing a combined score.

Setting attainable thresholds for the individual criteria is crucial because it enables all aspects of interest to be considered equally in the model evaluation. The logit transformation allows the criterion values to be placed on a common metric and the differences around the inflection point (i.e., cutoff values) to be maximized. If the thresholds are set too ambitious, this might lead to a criterion being included with (close to) zero in the overall pheromone level and effectively not being considered in the model evaluation. Determining empirical thresholds based on the available data and choosing flat(ter) slopes can avoid this problem. Thus, although ACO is an algorithmic approach for test assembly, some parameter tuning remains necessary to achieve optimal results.

## Future applications and investigations of ACO for simultaneous assembly of multiple tests

Future studies might investigate alternative practical use cases of ACO for simultaneous test compilation and the formal limits of the algorithm in test compilation. In the present study, we optimized a set of criteria we deemed relevant for parallel knowledge tests that can be used interchangeably for general-purpose applications. Instead of interchangeable parallel tests, tests could also be designed to optimally track a learning process in an educational context or to maximize predictive validity in a selection context. For example, multiple scales with a common set of linking items and increasing difficulty of the remaining items might aid in adequately mapping learning progress. Such a linking design and increasing difficulty can be implemented in the optimization process via constraints on the selected items and mean shifts in the test characteristic curves across test versions.

Alternatively, in a selection context, it may be of interest that the different test versions are maximally and equally predictive of a particular criterion measure. In a stepwise selection process (e.g., first a short online pretest for preselection, then a comprehensive onsite test), ACO could compile tests that maximize information gain or incremental predictive validity at a specific point in testing (Feng & Hancock, 2021). Such an approach with sequential tests would be similar to multistage testing (Yan et al., 2014). Multistage tests are better suited to maximizing measurement accuracy over the entire ability range. However, they cannot be easily implemented in common survey platforms and are also much more limited in terms of the criteria that can be optimized; that is, they are typically limited to test-inherent criteria such as reliability or difficulty. With ACO, a sequential set of tests can be compiled that considers a multitude of criteria, several of which we have already studied (e.g., fairness, criterion correlation). Therewith, the capabilities of ACO go beyond other approaches typically implemented in functional programming (Breithaupt & Hare, 2015; van der Linden, 2005).

From a methodological perspective, simulation studies should more thoroughly investigate under which conditions ACO succeeds in identifying an optimal solution and how to improve the likelihood thereof. Formal aspects of the procedure, such as the number of ants and iterations, are straightforward to manipulate and quantify. However, the composition of the initial item pool (i.e., size, coherence, psychometric characteristics) will largely determine whether an optimal test form can be drawn. Simulating these aspects requires extensive knowledge about the constructs under consideration and the items used to study them. For example, in knowledge assessment, one would need to vary the dimensionality of the initial item pool (e.g., Steger et al., 2019), group differences (e.g., Schroeders et al., 2016b; Watrin et al., 2023), correlations with covariates (e.g., Ackerman, 1996; Cattell, 1987) and item-level correlations (i.e., within-item multidimensionality; Schroeders et al., 2021). However, all these phenomena have been insufficiently studied so far. Until well-founded empirical findings allow substantial simulation studies, ACO, combined with the proposed random sampling procedure, will allow researchers to pragmatically identify (close to) optimal solutions in a heuristic, data-driven way.

## Conclusion

We strongly encourage the application of ACO in parallel test assembly because it is a versatile tool that greatly facilitates complex test compilation under multiple constraints. We argue that ACO is excellent for bringing the item sampling aspect of psychological measurement more to the forefront, whether to support pragmatic questionnaire development as in the present study or to answer substantive questions.
